# Statistical Properties and Predictability of Extreme Epileptic Events

**DOI:** 10.1038/s41598-019-43619-3

**Published:** 2019-05-10

**Authors:** Nikita S. Frolov, Vadim V. Grubov, Vladimir A. Maksimenko, Annika Lüttjohann, Vladimir V. Makarov, Alexey N. Pavlov, Evgenia Sitnikova, Alexander N. Pisarchik, Jürgen Kurths, Alexander E. Hramov

**Affiliations:** 1 Neuroscience and Cognitive Technology Laboratory, Innopolis University, 1 Universitetskaya str., 420500 Innopolis, The Republic of Tatarstan, Russia; 2 University of Münster, Institute of Physiology I, Münster, 48149 Germany; 3 Yuri Gagarin State Technical University of Saratov, 77 Politechnicheskaya str., 410054 Saratov, Russia; 4 Institute of Higher Nervous Activity and Neurophysiology of Russian Academy of Science, Moscow, Russia; 5 Center for Biomedical Technology, Technical University of Madrid, Campus Montegancedo, 28223 Pozuelo de Alarcón, Madrid, Spain; 6 Potsdam Institute for Climate Impact Research, 14473 Potsdam, Germany; 7 Department of Physics, Humboldt University, 12489 Berlin, Germany; 8 Biological Faculty, Saratov State University, Saratov, 410012 Russia

**Keywords:** Scientific data, Epilepsy

## Abstract

The use of extreme events theory for the analysis of spontaneous epileptic brain activity is a relevant multidisciplinary problem. It allows deeper understanding of pathological brain functioning and unraveling mechanisms underlying the epileptic seizure emergence along with its predictability. The latter is a desired goal in epileptology which might open the way for new therapies to control and prevent epileptic attacks. With this goal in mind, we applied the extreme event theory for studying statistical properties of electroencephalographic (EEG) recordings of WAG/Rij rats with genetic predisposition to absence epilepsy. Our approach allowed us to reveal extreme events inherent in this pathological spiking activity, highly pronounced in a particular frequency range. The return interval analysis showed that the epileptic seizures exhibit a highly-structural behavior during the active phase of the spiking activity. Obtained results evidenced a possibility for early (up to 7 s) prediction of epileptic seizures based on consideration of EEG statistical properties.

## Introduction

Extreme events are rare significant deviations of a system variable from its mean value. This fundamental phenomenon is inherent in many real-life systems and manifests itself as rogue waves in the ocean, extreme rainfalls, financial crisis, traffic jams, monster blackouts in power grids, etc.^1–4^. From the physical point of view, a study of extreme events is useful for revealing hidden underlying mechanisms responsible for abnormally large fluctuations. The knowledge of these mechanisms can help in the development of efficient methods for predicting and controlling the system’s extreme behavior.

Extreme events were observed and extensively studied in many deterministic and stochastic systems. Different scenarios of the emergence of extreme events have been discovered in model equations, including coupled oscillators and complex networks^5–8^, and evidenced in several physical experiments with fluids, nanophotonics and optical systems^9–15^. Sudden climatic changes, epidemics and epilepsy^16–21^ have recently received significant attention from the viewpoint of extreme event theory.

In this work, we focus on epilepsy as a clinical manifestation of extreme events characterized by a recurrent and sudden malfunction of the brain caused by excessive and hyper-synchronous neuron activity in the brain. Almost 50 million people are currently suffering from this disease, which can put the individual’s life at risk due to recurrent and sudden incidence of seizures, loss of consciousness and motor control^22^. Modern medicine is only able to improve the state of about two thirds of the patients, and surgery can help very few of them. However, no therapy can help one quarter of epileptic patients. Therefore, the prediction of epileptic seizures can greatly improve the life quality of these patients and open new therapeutic possibilities^23,24^. Furthermore, the solution of this challenging and still open problem would provide benefits ranging from pure fundamental ones, related to the understanding of epileptic seizure origin, to the application of methods for seizure forecasting and control.

In this paper, we consider a special form of epilepsy known as *absence epilepsy* characterized by the occurrence of spontaneous seizures in the form of spike-wave discharges (SWDs) in cortical and thalamic EEGs^25^, which is extremely difficult to predict. We apply the extreme event theory to the analysis of statistical properties of epileptic brain activity of rats with a genetically predisposition to absence epilepsy recorded with electroencephalography (EEG). These rats exhibit several hundred spontaneous SWD per day and have a high face and predictive validity to human condition^26^. The discovered well-pronounced extreme event features of the electrical brain activity provide a possibility for early prediction of epileptic seizures using clinical monitoring and real-time EEG processing. Since in humans the thalamic region is not easily accessible for EEG measuring, the epileptic early-warning signal can be recorded from the cortical area only. The animal models can be easily extrapolated to humans because the mechanisms for absence epilepsy in humans and rats are very similar^27^. Indeed, there exists a well-validated genetic animal model of absence epilepsy in WAG/Rij rats^28,29^, which can be easily supplied with intracranial electrodes to record epileptic brain activity.

## Methods

### Experimental procedure

The study was done in 5 male WAG/Rij rats, three of them aged 9 months, and two 11 months. Animals were born and raised at the Institute of Higher Nervous Activity (Moscow, Russian Federation). The experiments were conducted in accordance with the EU Directive 2016/63/EU for animal experiments and approved by the Ethical Committee of Institute of Higher Nervous Activity. Prior to surgery rats were housed in small groups with free access to food and water and were kept at natural lighting conditions. After surgery rats were housed individually. Distress and suffering of animals were minimal.

The recording EEG electrode was implanted epidurally over the frontal cortex (AP +2 mm and L 2.5 mm relative to bregma). Ground and reference electrodes were placed over the cerebellum. The EEG signal that was constantly recorded in freely moving rats during 24 h, was fed into a multi-channel differential amplifier via a swivel contact, filtered by a 0.5–200 Hz band-pass filter and digitized with 400 samples/s per channel.

After experimental procedure experienced neurophysiologist manually marked SWD onsets in recorded 24 h EEG signals of all five rats. Onset is defined as time moment, when the first well-developed “spike” appears. The “spikes” along with the “waves” are distinctive features of SWD. Each “spike” appears as single oscillation with frequency of 7–8 Hz, extremely high amplitude and well-pronounced asymmetry. Thus, appearance of the first “spike” marks onset of SWD and the last “spike” corresponds to offset of SWD.

### Time-frequency analysis

For description of pathological brain activity in terms of extreme behavior, we used a time-frequency representation of the original rat’s EEG via a continuous wavelet transform (CWT), a suitable tool for neurophysiological data analysis^30^. CWT convolves the EEG signal *x*(*t*) with the basic function *ψ*(*η*) as

1 $$W(f,{t}_{0})=\sqrt{f}{\int }_{-{\rm{\infty }}}^{+{\rm{\infty }}}x(t){\psi }^{\ast }(f(t-{t}_{0}))dt,$$

where ‘*’ stands for complex conjugation. As a basic complex function of CWT, we used the complex Morlet wavelet

2 $$\psi (\eta )=\frac{1}{\sqrt[4]{\pi }}{e}^{j{\omega }_{0}\eta }{e}^{-\frac{{\eta }^{2}}{2}},$$

where *ω*_0_ = 2*π* is the wavelet central frequency. In our statistical analysis, we deal with normalized wavelet energy *W*_*n*_ = *W*/W^*^, where *W* is an original value of wavelet energy obtained from (1), and W^*^ is a 99.9th percentile of wavelet energy PDF during normal activity.

In our research we considered frequency range of 2–20 Hz. We chose this particular range since it includes all important SWD-related frequency components: main frequency of SWD (7–8 Hz), its first harmonics (14–16 Hz), possible preictal activity (2–4 and 5–9 Hz)^30^.

The wavelet analysis of EEG recordings was done using home written C/Cuda software for increasing computation performance^31,32^.

## Results and Discussion

**Figure 1:**
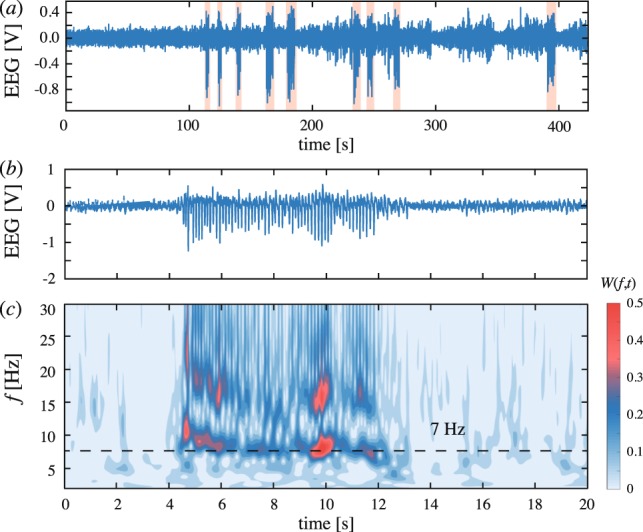
(**a**) Typical long-term EEG recording fragment of WAG\Rij rat containing a sequence of seizures (extreme events), highlighted with red color. (**b**) Typical EEG epoch with spike-wave discharge observed in epileptic brain of WAG\Rij rat. (**c**) Wavelet image of EEG segment with spike-wave discharge. Here, dashed line corresponds to 7-Hz oscillations wavelet energy.

SWDs are known to be an abnormal form of brain activity originated from hyper-synchronization in the cortico-thalamo-cortical neuronal network^33,34^. It is visually detected in long-term EEG recordings as an abrupt appearance of large-amplitude oscillations (Fig. 1(a)). Unlike typical extreme events, manifested as a short-term deviation of a measured variable from its normal state, individual SWD represents a regular sequence of spikes having a well-pronounced frequency (Fig. 1(b)). Thus, we refer SWD to as a single temporally distributed extreme event.

As can be seen from Fig. 1(c) which displays the time-frequency image of the corresponding EEG segment, SWD manifests itself as a sharp increase in the wavelet energy in the range of the main frequency (6–9 Hz) and its second harmonic (12–18 Hz). At the same time, the level of wavelet energy in the low-frequency range (<6 Hz) does not significantly change, as compared to the normal state. Due to the relation between wavelet energy in a particular frequency range and a size of neuronal population involved into particular rhythmic activity^35,36^, we concluded that during SWD, the majority of neurons located in the area of the EEG electrode implantation are in a synchronous bursting regime at 6–9 Hz and 12–18 Hz.

**Figure 2:**
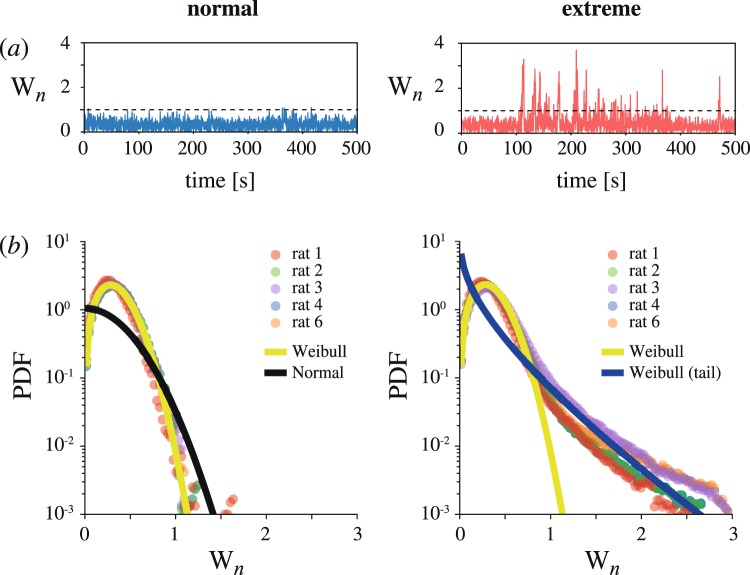
(**a**) Long-term time series of wavelet energy amplitude at main SWD frequency (7 Hz) during normal background (left panel) and pathologic activity (right panel). Here, dashed line indicates threshold value W^*^ corresponding to maximal level of wavelet energy during normal activity (see subsection Time-frequency analysis). (**b**) Semi-log PDF plots of wavelet energy amplitudes during background (left panel) and pathologic activity (right panel). The colored circles correspond to PDFs obtained from the experimental data of all five rats (bin size equals to 0.03). The solid curves are Weibull PDFs well-fitted to normal activity (yellow) and extreme tail (dark blue) with *p* > 0.99 tested via Pearson’s chi-squared test for each rat. Note, that background PDF is badly fitted by normal distribution (black curve with *p* < 0.01 via Pearson’s chi-squared test for each rat).

In the particular case presented in Fig. 1(a–c), the main SWD frequency is approximately 7 Hz. Considering the long-term time evolution of wavelet energy (Fig. 2(a)), one can note the difference between normal (left panel) and pathological (right panel) brain dynamics at the typical SWD frequency (7 Hz). Figure 2(b) displays the probability density functions (PDFs) of wavelet energy obtained from experimental data along with fitted distributions corresponding to normal (left panel) and pathological (right panel) behaviors. Surprisingly, we have uncovered a counter-intuitive fact that PDF of wavelet energy in case of normal EEG is not subject to Gaussian distribution with *p* < 0.01 via Pearson’s chi squared test (black curve in Fig. 2(b)). Instead, it is perfectly fitted by unimodal Weibull distribution (shape parameter *b* > 2)

3 $${f}_{W}(W|a,b)=\frac{b}{a}{(\frac{W}{a})}^{b-1}{e}^{-{(W/a)}^{b}}$$

with scale parameter *a* = 0.395, shape parameter *b* = 2.14 and *p* > 0.99 via Pearson’s chi squared test (yellow curve in Fig. 2(b)). Note that the perfect fitting of unimodal Weibull distribution to normal activity PDF is observed for each spectral component in the considered frequency range. It is known, that Weibull distribution well describes a particle size distribution obtained during fragmentation and performing geometric scale invariance (fractal properties)^37^. Due to this fact and taking into account the relation between wavelet energy and a size of synchronized neuronal population, we concluded that the process of formation and destruction of coherent clusters in brain cortex during normal activity is not random. On the contrary, it is likely to exhibit well-pronounced structural properties.

**Figure 3:**
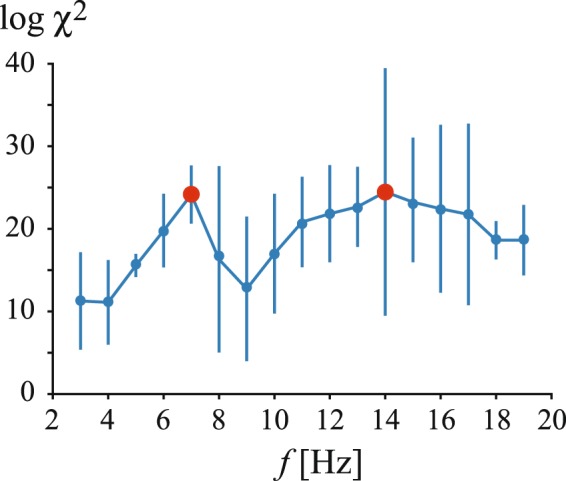
Semi-log dependence of Pearson’s chi squared statistics value on oscillation frequency. Here, *χ*^2^ quantifies the difference between wavelet energy PDFs associated with normal and pathological activity. Red dots indicate spectral components with maximal values of *χ*^2^, which are characterized by the highest degree of extreme behavior.

Compared to a normal state, pathological brain activity results in a well-developed heavy tail in the wavelet energy PDF. To validate the fact that the long tail is associated with the extreme behavior, we applied the extreme value theory, namely, the Pickands-Balkema-de Haan theorem^38,39^, and showed that the elongated tail can be fitted by the heavy-tailed Weibull distribution (shape parameter *b* < 1) with parameters *a* = 0.27, *b* = 0.73 and *p* > 0.99 via Pearson’s chi squared test (dark blue curve in Fig. 2(b)). Note that goodness of tail-fitting was tested in the range of *W*_*n*_ > 1. At the same time, the wavelet energy PDF corresponding to pathological brain activity is badly fitted by the unimodal Weibull distribution with *p* < 0.01 via Pearson’s chi squared test. In this case, the value of *χ*^2^ statistics provides the measure of the extremal behavior. Analyzing the dependence of *χ*^2^ on the oscillation frequency averaged over the group of participating rats (Fig. 3), one can observe two well-pronounced maxima marked with red dots. These maxima associated with the most extremal behavior correspond to the main SWD frequency (7 Hz) and its second harmonic (14 Hz). Notable, that for *f* < 6 Hz, 8 Hz <*f* < 10 Hz and *f* > 18 Hz the extremal properties are less pronounced. Thus, the statistical analysis demonstrates that abnormal brain activity related to absence epilepsy seizures exhibits well-pronounced properties of extreme dynamics. This type of behavior is localized in particular spectral ranges conditioned by the main frequency and its second harmonic.

**Figure 4:**
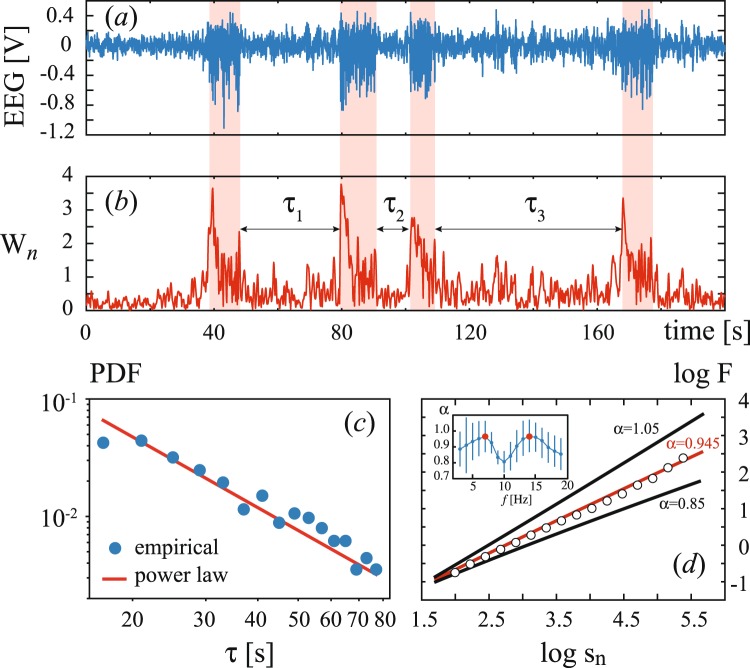
(**a**) Segment of EEG containing a sequence of SWDs and (**b**) corresponding wavelet energy time evolution. The arrows in (**b**) indicate recurrence times between neighboring SWDs. (**c**) Log-log PDF plot of return times collected from experimental recordings of all five WAG\Rij rats (blue circles with bin size of 4 s). The solid red line corresponds to power-law distribution *p* ~ *τ*^−*γ*^ with *γ* = 3/2 (*p* > 0.99 via Pearson’s chi squared test). (**d**) DFA analysis for 7-Hz wavelet energy. Here *s*_*n*_ = *s*/Δ is a normalized time window with Δ = 2.5 ms. The circles show log*F*(*s*_*n*_) calculated from experimental data and averaged over all participating rats. The insert shows the frequency dependence of slope *α*.

It is known^40^ that during active behavior phases, arousal and deep slow-wave sleep SWDs are rare, because the characteristic interval between subsequent seizures lies in the range from several tens of minutes to one hour. On the contrary, sequences of SWDs with short return times are observed during a state of drowsiness or passive wakefulness (Fig. 4(a,b)). The problem of regularity and interrelation of absence seizures during these stages is of undoubted interest. Despite its close association with the vigilance state, SWD has long been thought as unpredictable in nature, occurred from apparently normal background EEG. Now, it is possible to assess these issues considering SWDs from the viewpoint of the extreme event theory.

To examine the clustering properties of absence epilepsy seizures, i.e., to find correlations in SWD sequences, we carried out a statistical analysis of the return time between adjacent discharges. Figure 4(c) shows the PDF of return intervals *τ* calculated for recording segments with dense SWD sequences observed in all 5 participating rats. In case of an uncorrelated SWD sequence, one expects that the return intervals are distributed according to the Poisson law. In turn, data correlation and long-term memory are determined by either stretched exponential or power law. As will be shown below, in a wide range of *τ*, the return time intervals of our data are power-law correlated (*p* ~ *τ*^−*γ*^) with *γ* = 3/2 (*p* > 0.99 via Pearson’s chi squared test). A good fit of the experimentally obtained return interval distribution by a power law is well-reproduced in the group of rats. This confirms that epileptic seizures during stages of a developed spiking behavior exhibit scaling properties and long-range correlations. Note, that our findings are in a good agreement with previous theoretical and experimental studies of intermittent behavior in epileptic brain^41–45^.

To prove the effect of data correlation observed in the PDF of return times, we apply the well-established technique, known as detrended fluctuation analysis (DFA)^46^, which allows studying a long-term evolution of wavelet energy *W*(*t*) in a wide range of frequencies. In long-term correlated data, the mean fluctuation *F*(*s*) of the signal in time window *s* obeys a power law log*F*(*s*) ~ *α* log*s*. Figure 4(d) displays the *F*(*s*) scaling, averaged over all experimental rats, observed in the wavelet energy time evolution of main frequency oscillations (7 Hz). It is seen that log*F*(*s*) is almost a straight line with the slope *α* = 0.945 in the log-log scale. As seen in the insert, the maximal slope, i.e., the maximal correlation occurs for 7-Hz and 14-Hz oscillations, that is well-correlated with the results of the statistical analysis presented in Fig. 3. Since these frequencies indicate dominant and subdominant SWD frequencies, the extreme behavior here is strongly pronounced. Thus, according to the return times analysis and DFA we can conclude that the rat’s epileptic brain exhibits a highly structural and self-organized behavior during dense spiking activity phases.

**Figure 5:**
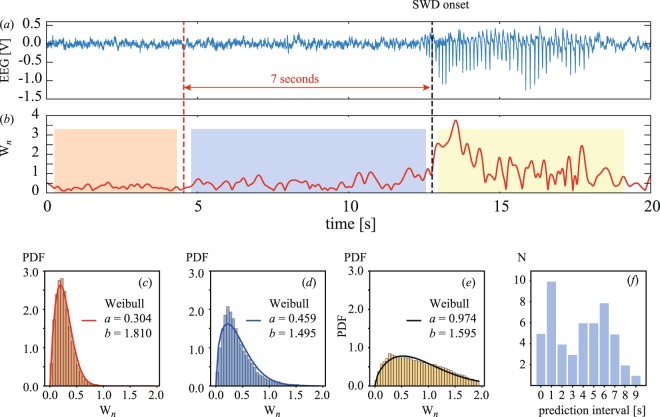
Illustration of SWD predictability. (**a**) Original EEG segment containing single SWD, (**b**) corresponding 7-Hz wavelet energy evolution. The black dashed lines indicates SWD onset. Time interval between red and black dashed lines is 7 s. (**c**–**e**) PDFs of segments marked in (**b**) by red, blue, and yellow; the solid lines in (**c**–**e**) are unimodal Weibull approximations. (**f**) Histogram of prediction intervals obtained for 50 seizures over all 5 participating rats (10 seizures for each animal).

The uncovered properties of a long-range correlation in the epileptic behavior are inherent to systems in the vicinity of a critical point^47^, where the system amplifies any fluctuations due to increasing instability. This effect known as prebifurcation signal (noise) amplification has been observed in physical, ecological and biomedical systems (see, e.g.^48–51^,). Notably, this effect is completely unobvious from original EEG recording, but clearly seen when considering wavelet energy evolution in a particular spectral range is associated with the main SWD frequency (Fig. 5(a,b)). To reveal this phenomenon in long-term epileptic EEG records, we considered distributions of the wavelet energy amplitudes of 7-Hz oscillations assessed within 10 randomly chosen EEG fragments from single rat recordings. Each fragment contained SWDs, which onsets had been manually marked by expert-neurophysiologist. From these fragments we collected 1-s epochs of ictal activity (1 s after onset), preictal activity (1 s before onset) and interictal activity far before onset (10 s before). Afterwards, we constructed wavelet energy PDFs corresponding to each type of brain activity across collected epochs (Fig. 5(c–e)). As seen from this figure, each type of brain activity is characterized by a specific form of Weibull curve. During normal brain activity, the wavelet energy variation is very low and the scaling parameter *a* of fitted unimodal Weibull distribution is also small (Fig. 5(c)). However, when a seizure approaches, the fluctuations of the wavelet energy increase along with *a*. It is seen that preictal activity clearly differs from normal and ictal activities, characterized by the highest variance and the scaling parameter *a* (Fig. 5(c–e)). It follows that the transition from normal brain activity to the seizure does not occur abruptly; but it is preceded by a well-defined precursor with distinctive statistical properties. Thus, the time interval of SWD prediction can be measured in a following way. The distribution of wavelet energies is constructed in a floating 1 second window and when it is well-fitted with known preictal PDF (Fig. 5(d)) the precursor is detected. The goodness of fit is tested via Pearson’s chi squared test (*p* > 0.9). Therefore, the time interval from precursor detection to SWD onset is the prediction interval. To verify the predictability of absence epilepsy seizures we have checked the prediction intervals for 50 epileptic events collected over all 5 participating rats (10 seizures for each animal). Corresponding histogram of prediction intervals is presented in Fig. 5(f). One can see, that prediction intervals are of 1–7 s. At the same time, 5 of 50 seizures have been either poorly predicted (up to 0.5 s) or detected on the onset.

The obtained results are related to the important problem of early prediction of SWD seizures^52^. According to the Review by van Luijtelaar *et al*.^53^ a considerable success has been achieved in the field of absence seizures detection during last years, yet the research on their prediction is not so fruitful. However, there is a number of successful attempts in this area. In particular, Li *et al*.^54^ have provided a predictability analysis of absence seizures via permutation entropy approach. They considered experimental EEG dataset of 28 rat (GAERS) containing 314 seizures, from which 169 have been predicted with average anticipation time of 4.9 s. Van Luijtelaar *et al*.^55^ have analyzed the origin of SWDs in WAG/Rij rats and discovered that absence seizures are preceded by Δ (1–4 Hz) and *θ* (4.5–8 Hz) precursors. Afterwards, Maksimenko *et al*.^34^ have developed a system for real-time absence seizure control based on detecting Δ and *θ* precursors. This system allows 45% of seizures to be predicted with anticipation time of 1–2 s. Also, Sorokin *et al*.^56^ have demonstrated the correlation between SWDs and preictal changes in *β* oscillations (20–40 Hz) 1.5 s prior seizure onset robust across different recordings, which seems to be relevant in developing new predictive algorithms.

In the context, results of our research are in agreement with mentioned studies on seizure prediction. We suppose, that possibility to predict seizures for 7 s interval is exciting, since it opens a way to prevent ongoing seizure by optogenetic or electrical brain stimulation, where early prediction is highly demanded^57,58^.

## Conclusions

To summarize, we have studied epileptic brain dynamics using the extreme value theory. We have shown, for the first time to the best of our knowledge, that during periods of spiking activity, the epileptic brain exhibits statistical properties of extreme events in the range of spike-wave-discharge (SWD) characteristic frequencies. It is notable, that uncovered statistical properties of SWDs are more in line with classical definition of extreme events, than with its special type – dragon-king behavior – as one may expect from numerical modeling of neuronal systems^59^. The detailed analysis of epileptic brain EEG recordings from the viewpoint of extreme events revealed self-organization properties of the brain’s spiking activity. In particular, we have found that return intervals between epileptic seizures obey a power law behavior in long-range correlations in the brain. By considering the brain as a dynamical system, we detected an increase in the fluctuation amplitude near a critical point preceding a seizure. The presented results open a new possibility for early SWD prediction by real-time tracing of the variance of the wavelet energy PDF.
